# CELLULAR GRAFTS IN MANAGEMENT OF LEUCODERMA

**DOI:** 10.4103/0019-5154.53194

**Published:** 2009

**Authors:** Venkataram Mysore, T Salim

**Affiliations:** *From the Venkat Charmalaya - Centre for Advanced Dermatology, Consultant Dermatologist, Bangalore, India; Senior Lecturer and Dermatologist*; 1 *From the Venkat Chamalaya - Calicut Medical College, Calicut, India*

**Keywords:** *Vitiligo*, *leucoderma*, *grafts*, *cellular grafts*, *melanocyte*, *transplantation*

## Abstract

Cellular grafting methods constitute important advances in the surgical management of leucoderma. Different methods such as noncultured epidermal suspensions, melanocyte cultures, and melanocyte-keratinocyte cultures have all been shown to be effective. This article reviews these methods.

## Introduction

Surgical methods in the management of leucoderma (both vitiliginous and nonvitiliginous) have a well-documented and established role in the management of resistant cases. The basic principle of all surgical methods is transfer of melanocytes from uninvolved skin into a stable leucoderma lesion, where they grow into, and function as, effective epidermal-melanin units. Although noncellular grafts such as, split thickness, suction blister, and punch grafts are the mainstay of surgical management, a number of cellular methods have become increasingly popular in recent times.[1]

Cellular grafts[2] include different methods such as, noncultured basal cell enriched melanocyte suspension, cultured mixed melanocyte-keratinocyte suspension (with or without carrier), and cultured pure melanocyte suspension. This article reviews these different cellular methods, their place in the management of leucoderma, their methodology, and relative advantages and disadvantages. It will also review the existing data on their relative efficacy and possible progress in the future.

## Cellular Grafts Versus Tissue Grafts

Although tissue grafts are the mainstay of surgical treatment of leucoderma at present, they have a number of disadvantages [Table 1]:[3,4]

1. The major disadvantage of tissue grafts is the need for a large amount of donor skin, equal to or slightly greater than the recipient area to be grafted. Obtaining such large grafts needs considerable surgical skill and may not be feasible in patients with large lesions, particularly in generalized vitiligo vulgaris.
2. Large donor grafts are also a cause of significant morbidity to the patients, as the donor area takes almost 10 days to heal.
3. Infection, postoperative pain, and scar in the donor area are the possible complications.
4. Application of grafts is time consuming, cumbersome, and difficult for small lesions, and over difficult areas such as, earlobe, umbilicus, fingertips etc.
5. Strict immobilization of the grafted area is needed after application of the tissue graft, which is cumbersome and inconvenient.
6. Postoperative results in case of tissue grafts are technique and operator-dependent. Several complications such as cobblestoning, perilesional halo, and hyper pigmentation of recipient area are common.
7. Hyperpigmentation of grafted skin is common and resistant to treatment.
8. Finally, some areas such as fingertips, do not “take” well and tissue grafting yields disappointing results in these areas.[3]

These disadvantages are sought to be overcome by cellular grafts. The following advantages have been claimed for cellular grafts [Table 1].[5]

1. The main advantage of cellular methods over tissue grafts is that a smaller amount of donor tissue is needed to cover a large recipient area. The donor melanocyte number can be potentially expanded to cover an area several times larger than the donor area. The donor:recipient ratio can be between 1:8 – 1:150, depending on the method used.
2. The small area of donor harvesting would reduce postoperative pain and morbidity.
3. Application of cell suspension is much easier when compared to the application of grafts, particularly in certain areas such as, ear, umbilicus, genitals etc.
4. It has also been suggested that the immobilization needed is less in case of cellular grafts.
5. Postoperative blending of the treated area with surrounding normal skin is said to be better.
6. Finally, cellular grafts may be more suitable for certain areas such as, fingertips, which have been conventionally regarded as difficult areas for tissue grafts.

These advantages explain the recent interest in these methods. However, it should be emphasized that transplantation by cellular methods, for treating vitiligo, is still at a relatively early stage of development. Standardization still needs to be achieved with some of the methods. Concerns have been expressed about possible tumourigenic potential in some methods. There has been limited data on the subject and only few centre perform these methods. Therefore, the methods await full evaluation. However, the scenario is changing rapidly with many centres using these methods and reporting their data.

**Table 1:** Comparison between tissue and cellular methods of grafting

Feature	Tissue grafting techniques	Cellular grafting techniques
Donor area	Need large amount of donor skin	Need small amount of donor tissue
Donor area morbidity	More	Less
Application	Time consuming and cumbersome; difficult over areas such as lips, ear lobes, scrotum, etc.	Easy, quick; can be applied to any area
Immobilization	Strict immobilization, needed for up to 5 days	Strict immobilization not needed
Postoperative complications	More	Less
Results	Technique oriented and common	Less common; technique oriented
Recipient area hyper pigmentation	Common and resistant to treatment	Less severe
Technique	Some techniques such as split thickness grafts need expertise and training	Need training and separate staff for preparation; prolonged learning curve
Infrastructure	Needs simple instruments	Needs chemicals, and in some methods, special equipment
Duration	Quick, particularly split thickness graft	Slow; culture methods take weeks
Cost	Cheaper	More expensive
Availability	Can be done anywhere	Only in few centres at present
Concerns	None	Methods still need standardization; long-term data limited for some methods
		Concerns about tumorigenic potential in some methods

### Indications[3,6,7]

Indications for cellular grafts are similar to those used for tissue methods. The commonest indication is stable vitiligo, refractory to standard medical and physical modalities of treatment. It is particularly useful as a primary treatment for segmental vitiligo and focal vitiligo. Stable vitiligo is defined by the task force of the Indian Association of Dermatologists, Venereologists, and Leprologists[6] as vitiligo that has been stable for the last one year, as per the following criteria; *a patient reporting absence of new lesions, absence of progression of existing lesions, and absence of Koebner phenomenon in the previous one year.* Spontaneous repigmentation and absence of Koebner's phenomenon are also considered as favorable signs for vitiligo surgery. Test grafting may be done in doubtful cases.[6]

Other indications include piebaldism, post-burn leucoderma, chemical leucoderma, nevus depigmentosus, and halo nevus.[7]

## Contraindications

Are few and include:

1. Bleeding disorders
2. Active vitiligo
3. Keloidal tendency

Age of the patient needs to be carefully considered while choosing a patient for surgery. There is no uniformly accepted age limit for surgery. It has also been seen that results of surgery are better in young patients than in older patients.[8,9] However, there are many limitations for performing tissue grafts in children.[6,7] Taking a large donor graft, its subsequent application, and immobilization of the recipient area would all be difficult in a child. Vitiligo surgery is usually performed under local anaesthesia, which would be difficult in children. General anaesthesia for vitiligo surgery in a young child would pose a higher degree of risk and would therefore be unacceptable. Finally, the progress of the disease in children is unpredictable, and therefore, stability is difficult to predict. As such, there are no published data with respect to cellular grafts in children. However, cellular methods, which require considerably smaller donor skin, are easier to apply and need less immobilization, would perhaps be more suitable and feasible in young adults and adolescents as compared to tissue grafts (authors' personal opinion). Hence, any decision to perform cellular grafting in young patients should be taken after considering all aspects of the disease, individual needs, and after proper counseling.

## Informed consent

As for any surgical procedure, informed consent should be obtained after detailed counseling. Counseling should include detailed information on the method to be used, possible variability of results, and relapse of disease. If culture methods are to be used, specific information about the lack of long-term data should be provided.

### Types of cellular graft methods

Different cellular methods include:[10,11]

- Autologous noncultured epidermal cell suspension
- Autologous cultured pure melanocyte transplantation
- Autologous cultured melanocyte - epidermal grafts

### Noncultured melanocyte grafting-epidermal suspensions

Autologous noncultured melanocyte-keratinocyte transplantation[12,13] (also called basal cell rich, noncultured melanocyte suspension) is the most commonly reported and also the simplest cellular grafting technique. Since this method is the most commonly used method among cellular grafts, it is dealt with in detail here.

## Basic principle of epidermal suspension

The basic principle is to separate the basal cells and the melanocytes by trypsinization, and prepare a suspension which is applied on the dermabraded lesional skin. This method allows a donor to recipient ratio of 1:5 or 1:10, and can therefore treat large areas with good results. It is also the simplest and the cheapest of all cellular methods and does not need expensive, sophisticated infrastructure. However, simple, specialized equipments such as, centrifuge, incubator, and reagents are needed. Training and experience are required to obtain standardized uniform results and hence there is an initial learning curve.

## Technique

Olsson and Juhlin first described a technique which has been modified subsequently and commonly followed.[14,15] The technique involves three essential steps:

1. Harvesting the donor skin of one-fifth to one-tenth the size of the recipient area, either by split thickness graft or suction blister grafting
2. Preparation of melanocyte rich basal layer cell suspension
3. Application of the cell suspension over the recipient area after preparing it by either dermabrasion or CO2 laser ablation.

## Equipments and consumables

The following chemical agents are needed:

1. Trypsin-EDTA solution of 0.25%, which is necessary to separate the dermis from the epidermis and also to free the keratinocytes
2. Trypsin Inhibitor, to neutralize excess trypsin
3. Dulbecco's Modified Eagle's Medium (DMEM)/ Nutrient Mixture F-12. 1:1 v/v 15 mmol/L HEPES buffer system. Various modifications of this medium have been used
4. Centrifuge to ensure proper suspension, after cell separation by trypsin
5. Aerobic incubator to ensure optimum conditions for the enzyme to act and also to ensure absolute sterilization
6. Micromotor diamond fraise dermabrader or a CO_2_ laser to de-epithelialize the recipient site
7. A manual or electric dermatome/suction apparatus to obtain grafts
8. Pasture pipette or calibrated micropipette to apply calibrated amounts of suspension
9. Two pairs of fine pointed forceps
10. Centrifuge tubes and petri dishes
11. Collagen dressings to apply over recipient area, after application of suspension

## Methodology

Obtaining donor: Donor graft is obtained by either split thickness or suction blister techniques.This step is described in detail elsewhere and hence will not be dealt here. It is advisable to administer a tranquillizer and preoperative antibiotic. The donor area is marked on the lateral aspect of the gluteal region or thigh, as per requirements of the given case. It is cleaned with povidone iodine and alcohol and anaesthetized by injection of 1% lignocaine. Application of eutectic mixture of local anaesthetics (EMLA) cream can help in reducing the injection pain and amount of lignocaine needed. A superficial skin sample is obtained using a skin grafting knife or a sterile razor blade on a straight artery forceps. The graft should be thin and uniform, so that obtaining cell suspension is easy and quick. In the authors experience, thick samples containing dermis are not suitable as it is more difficult to tease out the dermis, and will therefore need more time for obtaining the suspension. Alternate methods include obtaining donor skin by the suction blister technique. The donor site is then covered with tulle dressing.

Modifications of this method, such as obtaining skin from hairy scalp which is rich in melanocytes have been described.[16]

## Preparation of cell suspension

Step 1: Trypsinization: The skin sample is immediately transferred into a petri dish that contains 8 ml of 0.25% trypsin - ethylenediamine tetraacetic acid (EDTA) solution. The sample is turned back and forth to ensure complete contact with the solution. It is finally placed with the epidermis facing upwards. The sample is then incubated in an aerobic incubator at 37^°^ C for 50 minutes. After incubation, the excess trypsin-EDTA solution is removed and about 5 ml of trypsin inhibitor is added, to stop further action of trypsin.

Step 2: Separation of dermis from epidermis: The epidermis and the dermis are separated by using a pair of non-toothed forceps. This may need repeated teasing and is easy if the trypsinization is adequate. Additional incubation may be needed if separation is not possible. The separated dermis, which looks whitish, is discarded. The epidermis is then transferred to a medium that is suitable for melanocyte suspension. The medium used is Dulbecco's Modified Eagles Medium (DMEM) with F-12 nutrient mixture, 1:1 v/v and a 15 mmoles/L HEPES Buffer system. This is used in all stages of cell separation. The epidermal sheet is further teased with a forceps, to obtain a uniform suspension.

Step 3: Centrifugation: The material is then placed in a centrifuge tube and centrifuged at 2000 rpm for 10 minutes. A cell pellet, rich in melanocyte and basal keratinocytes is seen to have formed at the bottom of the tube. It is brownish in color. Pieces of epidermis, if present, are discarded. If separation is not proper, centrifugation may need to be repeated. The supernatant fluid is discarded and the pellet is resuspended in a total volume of 0.8 ml of fresh DMEM medium in a 1 ml syringe. To confirm the presence of melanocytes, a drop of suspension is placed on a slide and observed under an invertor microscope. In the experience of the authors, this is needed only in the initial learning stage. An alternative method to confirm the presence of melanocytes includes a phase contrast microscope.

The entire process of the preparation of cell suspension takes about 60 – 90 minutes. During this time, it is preferable for the patient to relax by watching television or by reading.

Transplantation: The recipient area is cleaned with povidone iodine and alcohol. While small areas can be treated under local anaesthesia, large areas, and in patients with poor compliance, general anaesthesia or nerve blocks are needed. Application of EMLA cream can help in reducing the injection pain and amount of lignocaine needed. Under local anaesthesia, using a high speed diamond fraise dermabrader, the recipient area is abraded down to the dermoepidermal junction. Other techniques such as CO_2_ ablation of the recipient area may also be used. The cell suspension is applied with a syringe or pipette and spread out evenly with its tip over the recipient area. The area is then covered with a thin transparent collagen film. Dressing is done with a sterile gauze piece, moistened with DMEM/F-12, and finally with Tegaderm (3M) dressing. It is important to ensure proper dressing, to prevent mobility, when lesions over mobile areas are being treated.

Postoperative care: Prophylactic antibiotics and analgesics are recommended for 1 week. Oral acyclovir may be considered in patients with a history of a recent episode of herpes simplex. Patients should be instructed not to move the transplanted area. The first 48 hours are crucial to ensure proper embedding of the melanocytes. There may be considerable oozing, with foul smelling discharge. The dressings are removed after a week.

**Figure 1:**
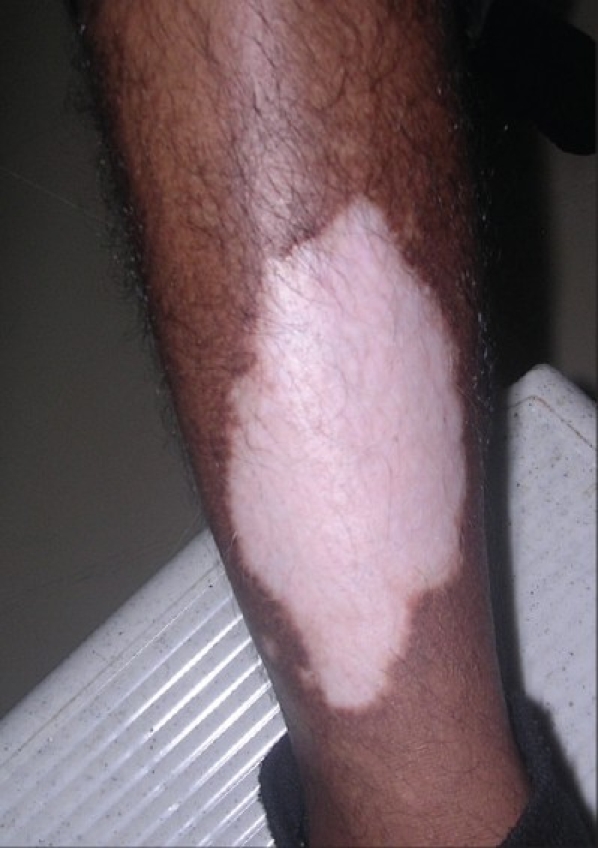
Vitiligo lesion on the leg before treatment

**Figure 2:**
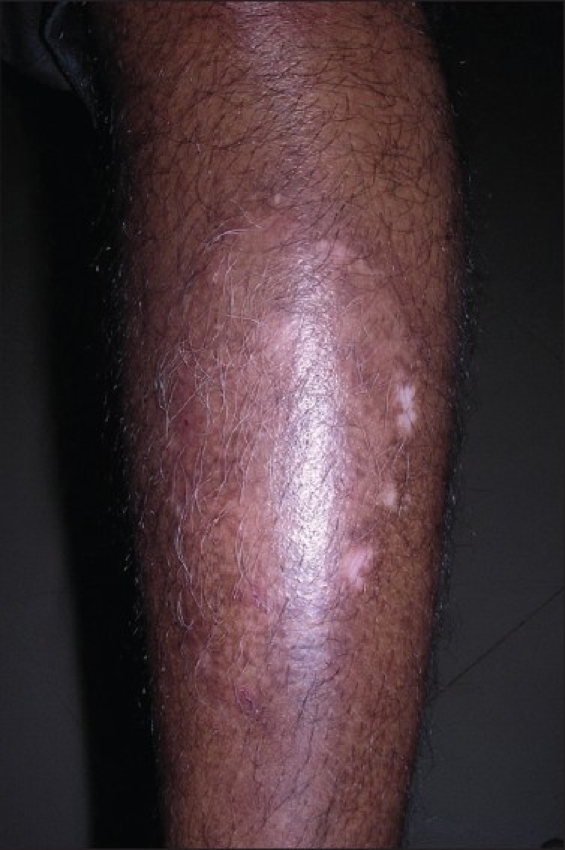
Vitiligo lesion on the leg after treatment with noncultured epidermal suspension

**Figure 3:**
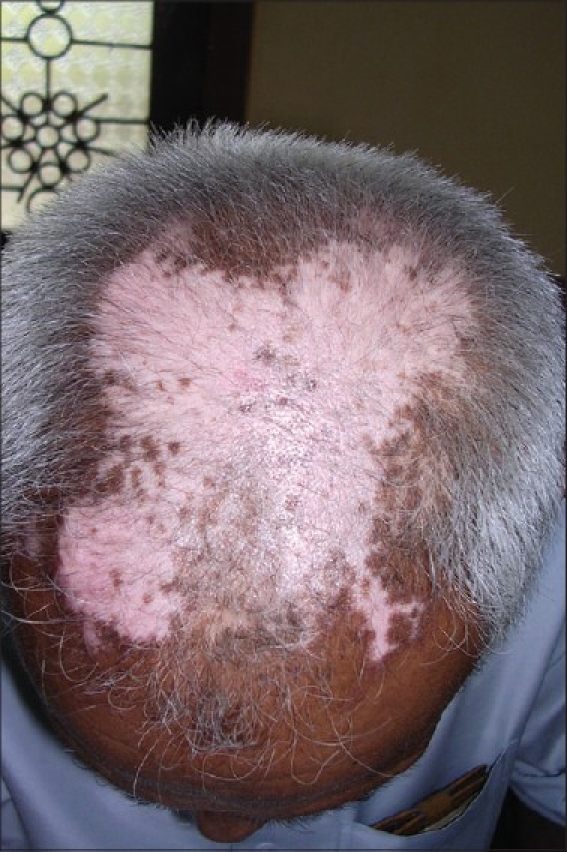
Vitiligo lesion on the scalp before treatment

**Figure 4:**
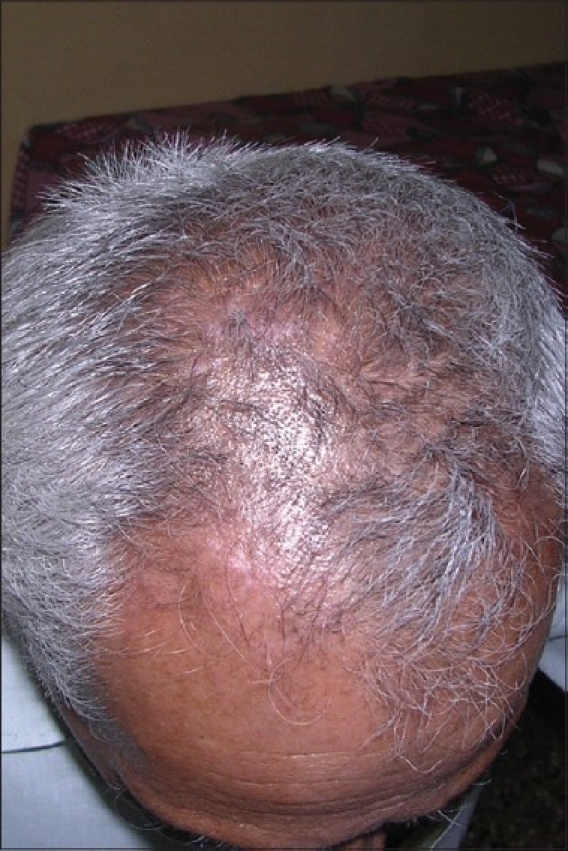
Vitiligo lesion on the scalp after treatment with noncultured epidermal suspension

**Figure 5:**
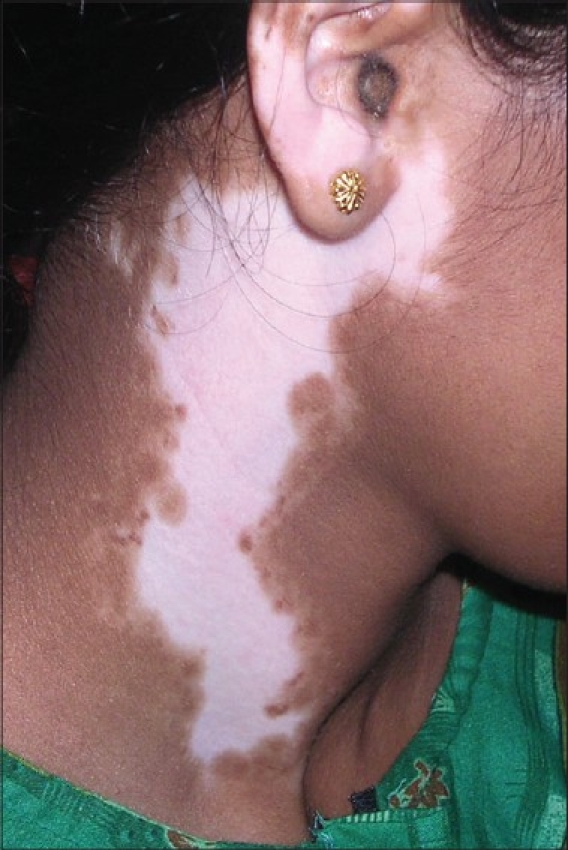
Vitiligo lesion on the neck before treatment

**Figure 6:**
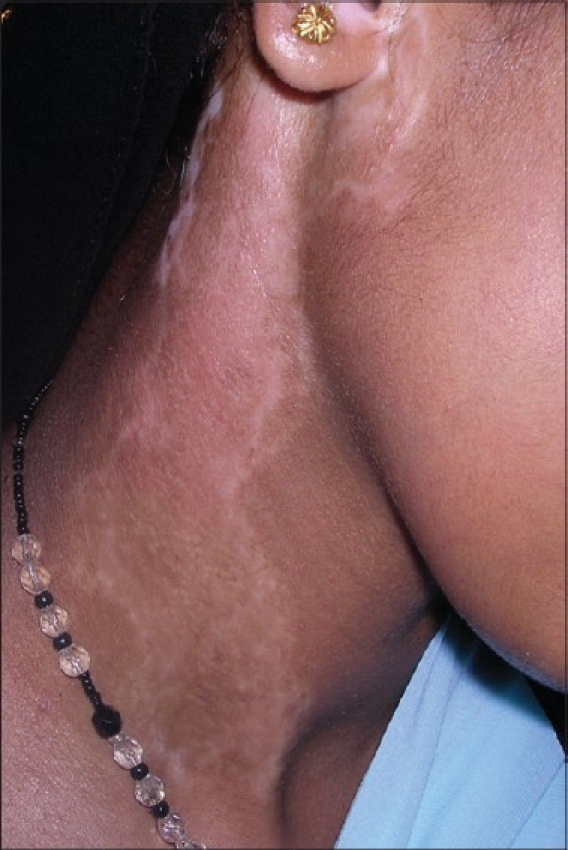
Vitiligo lesion on the neck after treatment with noncultured epidermal suspension

Complete epithelialization takes 6 to 10 days, depending on the site and extent of the area treated. Pigmentation usually starts within 4 weeks of grafting. Phototherapy can be started after 3 weeks following the procedure, and pigmentation is usually complete within 3 to 6 months [Figures 1–6].

## Disadvantages of the Epidermal Suspension Method

While it is a simple procedure, it has certain disadvantages too:

1. It requires specific cell culture grade biochemicals and media
2. The operating room should be equipped with an incubator, centrifuge, and sterile environment for preparation of cell suspension
3. It is more expensive than the tissue methods
4. There is a learning curve for the procedure. It takes experience to obtain a good suspension
5. It is time consuming

## Autologous Cultured Melanocyte Transplantation

In this method, *in vitro* cultured melanocytes are used in the surgical treatment of vitiligo.[17–20] The donor skin is obtained from the gluteal region by shave excision. The dermis is separated from the epidermis by trypsinization. Melanocytes and keratinocytes are dissociated by vigorous vortexing and seeded in a modified melanocyte medium containing growth factors. The melanocytes are cultured *in vitro* for 15 to 30 days by the addition of media and growth factors. Once sufficient numbers are achieved, the melanocytes are detached from the culture plates. The recipient area is prepared by superficial dermabrasion or CO_2_ laser ablation. The suspension is transplanted on the denuded recipient area in a density of 1000 – 2000 melanocytes/mm^2^. Pigment cells are transplanted either as a free suspension or with keratinocytes as a co-culture or integrated into the epidermal sheets. The site is secured with a gauze, soaked in culture medium, followed by an occlusive dressing. The patient is advised bed rest for 8 to 10 hours and the dressing is changed after 1 week.

The method can treat very large areas, and with a donor to recipient ratio of 1:20 – 1:30. Expansion of even up to 1:150 has been reported. An example of the medium used for cell culture is Hu16 medium, which consists of Ham's F12 nutrient mixture (Gibco) supplemented with 50 Î¼ g/mL gentamicin, 20 ng/mL recombinant human basic fibroblast growth factor, 20 Î¼g/mL isobutylmethylxanthine, 10 ng/mL cholera toxin, and 20% fetal calf serum. The cultures are incubated at 37°C in a humidified 95% air and 5% carbon-dioxide atmosphere.[17]

However, there have been some safety concerns about the use of cultured autografts in vitiligo. Mutagenecity and possible risk of cancer have been cited as potential risks. 12- tetradecanoylphorbol 13-acetate (TPA) had been previously used in the culture medium, and since this is a tumor promoter, concerns about its long-term safety have been expressed. Nevertheless, the recent availability of TPA-free and serum- free media is a possible solution to this problem. Beta fibroblast growth factor (bFGF) has been used as a substitute and is believed to provide satisfactory result.[17,18] The presence of cyclic adenosine monophosphate elevating agents (such as isobutylmethylxanthine and cholera toxin) is also essential for the growth of melanocytes in the absence of TPA.[17]

## Autologous Cultured Melanocyte-Epidermal Grafts

In this technique,[21–27] the cells are seeded in a medium that allows co-cultivation of keratinocytes and melanocytes. Few weeks later, a cultured epidermal sheet is obtained, released by dispase and attached to a petrolatum gauze. The recipient site is prepared as described in cultured melanocyte transplantation. The gauze is applied on the recipient site and dressed. The major advantage is that cells expand in the cell culture to treat a large area.

### Analysis of results by different cellular grafting techniques

Several recent publications have documented the efficacy of different cellular methods. Pigment production, long-term results, and their relative efficacy in difficult areas have all been examined.

In an important study,[28] 132 patients were treated with different types of grafts, such as, tissue grafts (ultrathin skin grafts), autologous cultured melanocytes, and basal layer suspension, on 176 occasions in total. They were followed up for 1 to 7 years after surgery and responses to different treatments were analyzed. Stable types of leucoderma, (segmental vitiligo and piebaldism) responded in most cases with 100% repigmentation, regardless of the surgical method used. In vitiligo vulgaris, the ultrathin epidermal sheet method gave a somewhat better overall result. The trunk, arms, and legs (not including elbows and knees) responded best. However, the outcome was not satisfactory in the knee and elbow areas. Fingers and elbows were the most difficult areas to repigment by any method. Slight hyperpigmentation was common when ultrathin epidermal sheets had been used. No scars or indurations were seen in the treated areas. The study concluded that melanocyte transplantations were the methods of choice in stable types of leucoderma, but progressive, widespread vitiligo vulgaris should never be selected for transplantation. The study emphasized the importance of the proper choice of method, according to the anatomical location to be treated.

Another study compared noncultured epidermal cell suspensions with placebo in a paired double blind trial.[29] A total of 33 paired, symmetrically distributed leukodermic lesions, all resistant to therapy, were treated in 28 patients. A significant difference between cellular grafts and placebo was observed after 3, 6, and 12 months (*P* <.001, *P* =.002, and *P* =.002, respectively). Transplantation resulted in repigmentation of at least 70% of the treated areas.

However, other studies have documented less than impressive results in lip-tip vitiligo.[30] Thirty-two patients carrying different types of vitiligo were treated with autologous cultured epidermal grafts. The average percentage of repigmentation over all areas, evaluated after 12 to 36 months of follow-up, was 77%. Independent of the type of vitiligo, average percentages of repigmentation of extremities and periorificial sites were 8 and 35%, respectively. Percentages of repigmentation of all other body sites ranged from 88 to 96%. The study concluded that cultured epidermal grafts could be considered a real therapeutic surgical alternative for “stable” vitiligo, but not for lip-tip vitiligo. Another study demonstrated satisfactory results in only 50% of lesions with both melanocyte-rich cell suspension technique and melanocyte culture (MC) technique.[31]

Long-term efficacy after epidermal suspension has been well established.[32] One hundred and forty-two patients with vitiligo vulgaris were treated and observed for a period of up to 6 years. Eighty (56%) patients showed excellent, 15 (11%) showed good, 13 (9%) showed fair, and 34 (24%) showed poor repigmentation, which was retained till the end of the respective follow-up period.

A more recent study, studied the results in difficult-to-treat areas, such as, fingers and toes, palms and soles, lips, eyelids, nipples and areolas, elbows and knees, and genitals.[33] In bilateral vitiligo, more than 65% repigmentation of the treated areas could be achieved in more than 50% of the patients. In unilateral vitiligo, 65% repigmentation of the treated area could be achieved. The study concluded that the concept of a “difficult-to-treat site” is a relative term and depends on the technique used. Noncultured melanocyte keratinocyte transplantation does not require any special precautions to treat these anatomical sites.

These promising results have led to the commercial availability of these methods. Of late, a product called Recell has been marketed.[34] Future possibilities, such as, obtaining melanocyte stem cells from hair follicles and further refinement, and simplification of the existing techniques look promising.

The recent advances in cellular methods and their relatively easier availability have indeed brightened the outlook for vitiligo patients. However, it has to be remembered that unless the precise cause for vitiligo is found and relapses can be prevented, a cure for this condition will remain elusive. Also, in view of the severe social stigma that the disease causes and the resulting psychological disability, what is statistically impressive may not be necessarily satisfactory to patients. Persistence of even a small lesion will continue to bother the patient. What the patients need and look for is complete (100%) repigmentation, without any relapse. It is a sobering thought that these objectives cannot be achieved by any of the existing methods.

## Conclusion

Cellular methods have obvious advantages in the surgical management of leucoderma, such as, requirement of small size of donor and efficacy in large lesions of vitiligo. However, the cost of the treatment and need for special reagents are disadvantages. Nevertheless, with continued refinement of this technique, they are bound to get more popularity in the years to come.
